# Woody species composition and diversity of riparian vegetation along the Walga River, Southwestern Ethiopia

**DOI:** 10.1371/journal.pone.0204733

**Published:** 2018-10-17

**Authors:** Misganaw Meragiaw, Zerihun Woldu, Vegard Martinsen, Bal Ram Singh

**Affiliations:** 1 Department of Plant Biology & Biodiversity Management, College of Natural and Computational Sciences, Addis Ababa University, National Herbarium, Addis Ababa, Ethiopia; 2 Faculty of Environmental Sciences and Natural Resource Management, Norwegian University of Life Sciences, Ås, Norway; Fred Hutchinson Cancer Research Center, UNITED STATES

## Abstract

The primary objective of this study was to examine the status of woody species composition and diversity along the Walga River of Wonchi, Southwestern Ethiopia. Fifty quadrats of 10 m x 50 m were laid at 500 m interval through systematic sampling method along the river line. Vegetation height (≥2.5 m) and DBH (≥2.5 cm) of only tree species were measured and altitude, ecological disturbances such as, grazing intensity and human impacts were included as main environmental variables at each of the sampled plots. The data was analyzed using different R statistical packages. Ninety-nine woody vascular plant species belonged to 81 genera and 45 familieswere recorded in Walga riparian vegetation. Only 10% of specieswere endemic to the Flora area. Asteraceae and Fabaceae had the highest number of species. Majority of the species (52.5%) were shrubs. Four major plant community types were identified: *Euclea divinorum*-*Maytenus arbutifolia* (1), *Pterolobium stellatum*- *Calpurnia aurea* (2), *Brucea antidysenterica-Prunus africana* (3), *Erica arborea-Hagenia abyssinica* (4). Species richness, true diversity and importance values were highestin community type 2(the lowest altitude ranges between 1976–2212 m a.s.l.) while evenness was highestin community type 3(mid altitude ranges between 2359–2676 m a.s.l.). Both community typeswere comprised of 56% of all recorded species and all endemic taxa except two. The highest percentage of species in lower frequency classes indicates a higher degree of floristic heterogeneity. There was a strong negative correlation (r = -0.65, p<0.001) between species richness and altitude with 42% of the variation in species richness per plot being explained by altitude. Our findings suggest that human disturbances and excessive livestock grazing are the main threats in community types1 and 2. We conclude that identifying major plant community types and underlying environmental conditions may help to manage and conserve forest resources in the area.

## Introduction

Plant communities grown along the river edges are known as the riparian vegetation, which is one of the fifteen terrestrial biomes of the earth [1,2]. Riparian vegetation is broadly defined as a terrestrial land with visible vegetation that interacts to permanent or temporary aquatic systems such as rivers, streams, lakes and wetlands [3–7]. Healthy riparian vegetation provide home for aquatic and terrestrial wild animals, water quality protection, stream bank stabilization, water capture and storage, as buffer zone to reduce flood and soil erosion, maintaining soil fertility and moisture for fast growth of plants [4; 8,9]. Because of such huge advantages, scientists and land managers are interested in understanding the status of riparian vegetation as key indicators to measure management practices [5,7–11]. Riparian vegetation isnaturally heterogeneous in the flora area with diverse species composition due to the change in microclimate, soil and land use types [6,12–14]. The variations of riparian vegetation are not only from region to region, but also shown along a single river line with altitude gradients and/or level of anthropogenic effects [15–17]. There are numerous perennial and flashing rivers and streams in Ethiopia, which support rich assemblages of plant species which respond differently to altitudinal variation [16,18–22]. Some of these are highlythreatened.

Over wide parts of the globe, many researchers agreed that disturbance in riparian vegetation is a complex interaction of both natural and anthropogenic effects [2,17,23,24]. In Ethiopia, the riparian vegetation coverissharply declining because of over exploitation of the natural forests at a rate higher than the natural regeneration. Expansion of agriculture, increase in livestock population, rapid expansion of invasive and exotic plant speciesand selective cutting tree for charcoal, fire-wood and construction are the proximal caused of the decline of vegetation cover and impoverishment of the species diversity [7,15, 23,25–27]. Although much is lost, the remnant flora of Ethiopia is still considered to be rich both in species diversity and endemism [22,26–29].

However, full description of the floristic composition and complete information on the status of species diversity in Ethiopian in general [1,12] and along the Walga riparian zone in particular is still lacking. This in turn poses some questions:

1. Are there consistent patterns of woody species diversity throughout altitude gradients along the river bank?
2. What are the relationships between the distribution of riparian plant communities and environmental variables (altitude and anthropogenic impacts)?
3. What is the implication of the current status of the riparian vegetation for conservation?

Keeping these questions in mind, the present study was aimed to: (1) examine the status of the woody species composition along the Walga River bank; (2) identify main plant community types; (3) assess association between altitude and anthropogenic impacts and riparian vegetation and (4) investigate major threats to the diversity of riparian vegetation and suggest bestmanagement practices.

## Materials and methods

### Description of the study area

**Fig 1:**
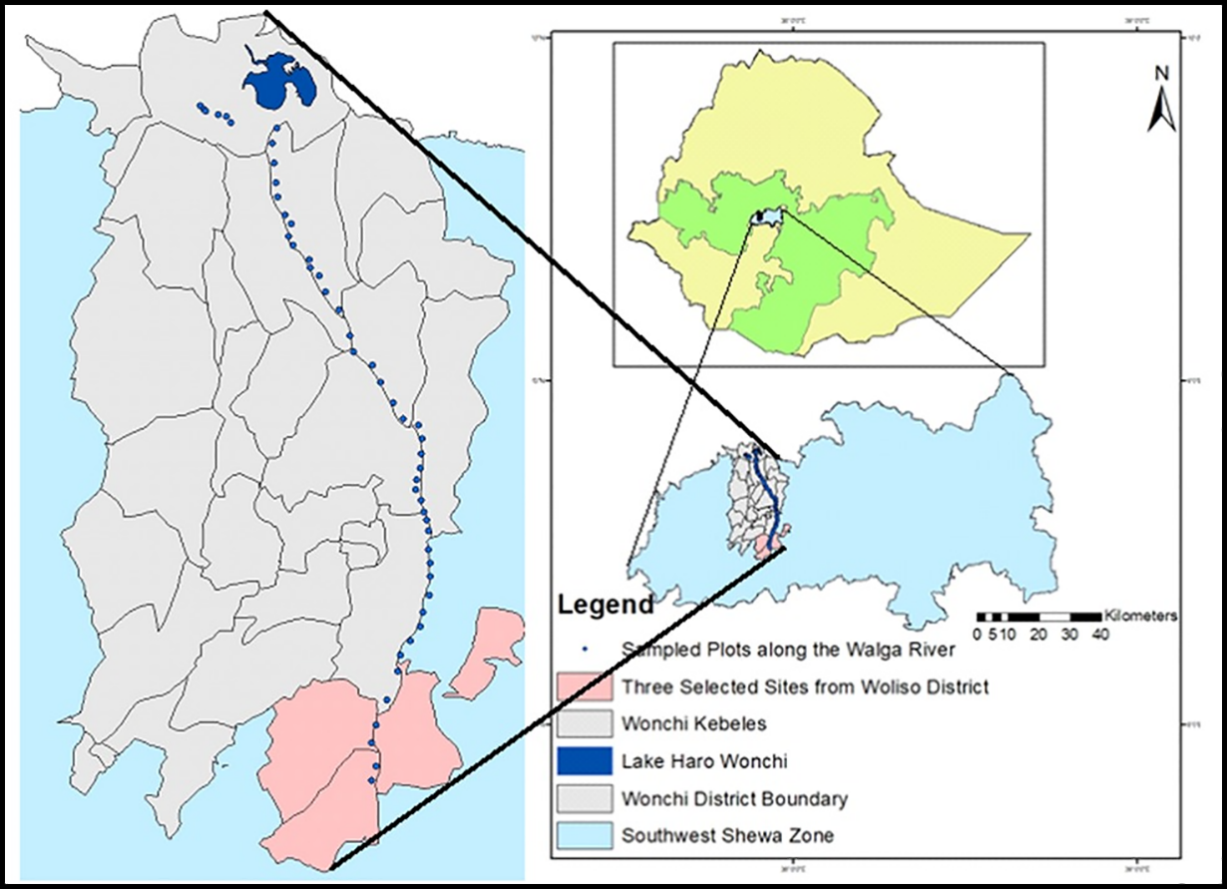
Map of Ethiopia showing Wonchi District and sample plots along the Walga River in Southwest Shewa Administrative Zone.

This study was carried out along the Walga Riverof Wonchi District, Southwest Shewa Administrative Zone of Oromia Regional State. Walga River crosses eight kebeles in Wonchi and three in Woliso(Fig 1). The altitude of the district rangesfrom 1700 to 3387 m a.s.l.The district covers an area of 474.56 km^2^ with a population of 119, 500. The dominant soil type is Luvisols with ashy parental material [30]. The district has both highland and midland types of agroecological zones along the river. The study area receives a uni-modal rainfall regime of up to 1129 mm of mean annual rainfall from 2004 to 2015. The longer rainy season is stretched from May to October. The mean annual temperature is 19.5°C with 12.4°C and 28.3°C minimum and maximum temperature, respectively (National Meteorological Agency, unpublished report from 2004–2015).

#### The role of riparian vegetation in Walga River

The vegetation type of the study area largely falls under the dry ever green montane forest [14]. The riparian vegetation also includes very small portion of subafroalpine vegetation at the peak. Walga River contributes countless services for the local community throughout the year, but the flow regime decreases in the dry season when people use the water for irrigation. The river is accessible starting from the HarroWonchi Crater Lake (major tributary among other three ones in Kibate Forest) until it mixes to Gilgel Gibe River. For instances, it has been used for irrigation systems to cultivate crops, different vegetables and fruit trees. Similarly, it harbors many wild edible and traditional medicinal plants. The riparian vegetation is also used as a home for diverse wild animals such as, velvet and colobus monkeys, hyena, leopard, rock hyrax, tree squirrel, antelope, Erckel’s francolin, goose, ducks and many bird species. Encroachments of the vegetation by the local people such as, selective cutting tree species for charcoal, fencing, construction, and firewood collection is rampart in the area. Expansion of agriculture, the spread of *Eucalyptus* plantation and intentional burning of *Erica* forests results in the expansion ofinvasive fern (e.g., *Pteridium aquilinum* (L.)).

### Floristic and environmental data collection

This study was conducted in Walga River. It is located in Wonchi District, Southwest Shewa Zone, Southwestern Ethiopia (Fig 1), 08^0^31.752'N; 037^0^55.894'E to 08^0^47.122'N; 037^0^51.796'E with altitude ranges from 1976–3078 m a.s.l. Specific permission was not required to conductthis study in the area where is not protected and the study did not involve extraction ofendangered species. However, a permitwas obtainedfrom Wonchi District Agricultural and Rural Development Office for plant specimen collections in the field based on the supporting letter from Addis Ababa University, College of Natural and Computational Sciences, Department of Plant Biology and Biodiversity Management.

The riparian vegetation data was collected through systematic sampling technique on both sides of Walga River bank with some portion of the flows from mid April to the mid of June, 2017. In total, 50 (10 m x 50 m each) quadrats were laid out at an interval of 500 m gap both sides of the river. Four plots were laid out downstream in the nearest three kebeles (Fig 1) to fill up the gaps of few unreachable steep areas. The present study was restricted to only tree and shrub growth forms for their bigger canopy size and dominant composition of the riparian zone. Herein, herbaceous plant species were not included because they were very rare in the river banks. One of the reasons may be the presence of over grazing and the seasonal growth of some species. All woody plant species within the quadrats were recorded with their local names.They were identified usingthe Flora of Ethiopia and Eritrea; and finally confirmed by experts before deposited at the national Herbarium (ETH).Height (≥2.5 m) and Diameter at Breast Height (DBH ≥ 2.5 cm) were measured at 1.3 m, as described by Woldu et al.[13] for 30 tree species only by using Suunto clinometers and diameter tape respectively. Individuals of the other shrub, both shrub and tree forms of species were not considered to this analysis because they were not qualified for the aforementioned height and DBH measures. Cover-abundance was estimated to all species following the modified 1–9 Braun- Blanquet scale [31].

Environmental data such as, altitude and geographic coordinates were recorded for each of the 50 quadrats using GARMIN GPS 72. In addition, ecological disturbances such as grazing and browsing intensity and human impacts (cutting tree for firewood, producing charcoal and burning of vegetation for expansion of agriculture) were noted as present or absent. The status of negative human interference was estimated through etic approach following Hadera [29] and 0–3 subjective scales are designated as: 0 = nil; 1 = low; 2 = moderate; and 3 = heavy. Likewise, livestock grazing intensity was estimated following Woldu and Backeus [32] as: 0 = nil; 1 = slight; 2 = moderate and 3 = heavy and 4 = destructive.

### Vegetation and environmental data analysis

The Walga riparian vegetation data were analyzed using different R statistical packagesincluding cluster, vegan and labdsv [33]. The number of plant community typeswas a compromise between the similarity in the sub-clusters (clusters within a single community type) and the dissimilarity between the clusters. Four plant communities were identifiedusing hierarchical clustering strategies withward's method and Euclidean distance. A synoptic table analysis was made to determine the characteristics species. Two characteristic species with high synoptic cover abundance values (mean frequency x mean cover-abundance) were used to name the plant community types following Kent [2] method. The relationship between species richness per plot and altitude was modeled by simple least squares regression. Represented by the equation: *y* = *mx* + *b* + ε Where, y is species richness per plot, m is slope or gradient, xis altitude and b is the intercept and ε is a random error component.All recorded species and transformed abundance scores were used as input data for Euclidean similarity matrix. The vegetation structure was described using frequency distribution, density, DBH, height and importance value index (IVI) for each species. Importance value index was computed for all woody species to determine their dominance position. Analysis of population structure for all 30 tree species was made on the basis of the five DBH classes (Class 1 = 2.5–10.0 cm, 2 = 10.1–20.0 cm, 3 = 20.1–30.0 cm, 4 = 30.1–40.0 cm, 5 = ≥ 40.1 cm). Height of the tree was calculated using a clinometers (percent scale) and tape measure: $Total\;height\;(m)=\frac{\left(Top\;measurement–bottom\;measurement\right)}{100}x\;Distance\;away\;from\;the\;tree\;(m)$. Diameter at breast height was also calculated as follows: DBH (cm) = Circumference (C)/π, where π = 3.14. The density of tree species and basal area (BA) of the vegetation were computed.Density, IVI and BA are calculated following Kent [2] method.

Shannon-Wiener diversity index (H) was used to analyze the species diversity. The equation is given as follows: $H=-\sum_{i=1}^{S}(Pi)(lnPi)$, Where S is total number of species in the community, Pi is the proportion or abundance of individuals of the i^th^ species and ln is natural logarithm. The Shannon's equitability or evenness (J) of the species was calculated as = H/ H_max_ = $\sum_{i=1}^{s}(Pi)(lnPi)/lnS$, where H_max_ = lnS. Species richness is the number of species in a given area. It is represented in equation form as $S=\sum_{i=1}^{S}Si$ = Σ Si, where Si is the number of individuals in the i^th^ species. Species richness is most often used in conservation studies to determine the sensitivity of ecosystems and their resident species.The index is not diversity by itself rather it can be converted into the effective number of species (true diversity) to make the diversity index number into more biological sense, which allows us to compare the biodiversity with other communities. The formal that converts the H into the true diversity (D) is given by following Jost [34] as follows: D = exp(*H*) which is the exponential of H. Community similarity was computed to evaluate the similarity between plant community types of the riparian vegetation in the study area, and with four other previous studies on the basis of their woody species composition. $Sorenson’s\;coefficient\;(CC)=\frac{2C}{S1+S2}$, where C is the number of species, the two communities have in common, S1and S2 is the total number of species found in community 1 and 2, respectively, was used to calculate the community similarity.

In addition to multivariate analysis of plant community,canonical correspondence analysis (CCA) was used to analyze the relationship between environmental data (altitude and anthropogenic impacts) and distribution of all recorded species in the plant communities. The raw data was transformed using vegtrans library to fit into ordination scatter plot with some syntax in ordination package of R (Vegan, CCA, labdsv). All sampled plots in response to three environmental data (livestock grazing and browsing, human disturbance and altitude) were included in this analysis.

## Results

### Species composition in Walga riparian vegetation

In total, Walga riparian vegetation is composed of 99 woody plant species belonging to 81 genera and 45 families. The majority of species were shrubs (52.5%), followed by trees with 30.3% while 17.2% of the species appeared as shrubs and trees at various sites. A complete list of all species with their author names is presented in S1 Appendix. All families are grouped under angiosperm except two families (Cupressaceae and Podocarpaceae) which are in gymnosperm group. Nearly one-third (32.3%) of the total species were found in three dominant families, Asteraceae, Fabaceae and Tiliaceae, consisting of 12, 11 and five species, respectively. The next dominant families, Celastraceae, Lamiaceae, Moraceae, Myrsinaceae and Rosaceae were represented each by four species and constituted 20.2% of the total species. The other five families which constituted 12 species were Anacardiaceae, Euphorbiaceae, Rubiaceae and Sapindaceae each with three species. The species of Capparidaceae, Ebenaceae, Ericaceae, Flacourtiaceae, Loganaceae and Solanaceae each were represented by two. The remaining 27 families were represented only by one species each.

Of the total plant species discussed herein, more than 10% were endemicto the Flora area.Nine species were endemic to Ethiopia while two were near endemic (which are confined to both Ethiopia and Eritrea). Except two species (*Crotalaria rosenii* and *Thymus schimperi*), nine occurred in community types2 and 3. With the level of threat category, eight species were of least concern (LC) while three species were assessed as near threatened (NT) (Table 1).

**Table 1:** Endemic species with their level of threat and distribution in the present community and Flora.

Species	Family	Habit	Community	IUCN category	Species distribution in the Flora Region
*Crotalaria rosenii*	Fabaceae	S	1	NT	SU, AR, BA, KF, SD
*Echinops longisetus*	Asteraceae	S	3	LC	GD, GJ, WU, SU, AR, WG, KF, GG,SD, BA, HA
*Erythrina brucei*	Fabaceae	T	2	LC	WU, WG, GJ, SU, BA, HA, IL, KF, GD, GG, SD
*Inula confertiflora*	Asteraceae	S	3	NT	WU, SU, AR, BA, HA
*Laggera tomentosa*	Asteraceae	S	3	LC	TU, GO, GJ, WU, SU, HA
*Lippia adoensis* var adoensis	Verbenaceae	S	2	LC	EE, TU, GD,GJ,WU,SU, AR,WG, KF, GG, SD, HA
*Maytenus addat*	Celastraceae	T	3	NT	SU, AR, SD, GG
*Millettia ferruginea* subsp. darassana	Fabaceae	T	2	LC	WG, SU, HA, BA, IL, KF, SD
*Rhus glutinosa* subsp. neoglutinosa	Anacardiaceae	S/T	3	LC	TU, WU, SU, WA, AR, BA, HA
*Solaneciogigas*	Asteraceae	S	3	LC	GD, GJ, WU,SU, AR,SD, IL, KF, BA, HA
*Thymus schimperi* subsp. schimperi	Lamiaceae	S	4	LC	EW, TU, GD, WU, SU, AR, SD, BA, HA

Note: Habit (T = tree, S = shrub); IUCN Threat categories (LC = Least Concern, NT = Near Threatened). Community type 1: *Euclea divinorum*-*Maytenus arbutifolia*, Community type 2: *Pterolobium stellatum*-*Calpurnia aurea*, Community type 3: *Brucea antidysenterica-Prunus africana*, Community type 4: *Erica arborea-Hagenia abyssinica*.

### Classification of plant communities in Walga riparian vegetation

**Fig 2:**
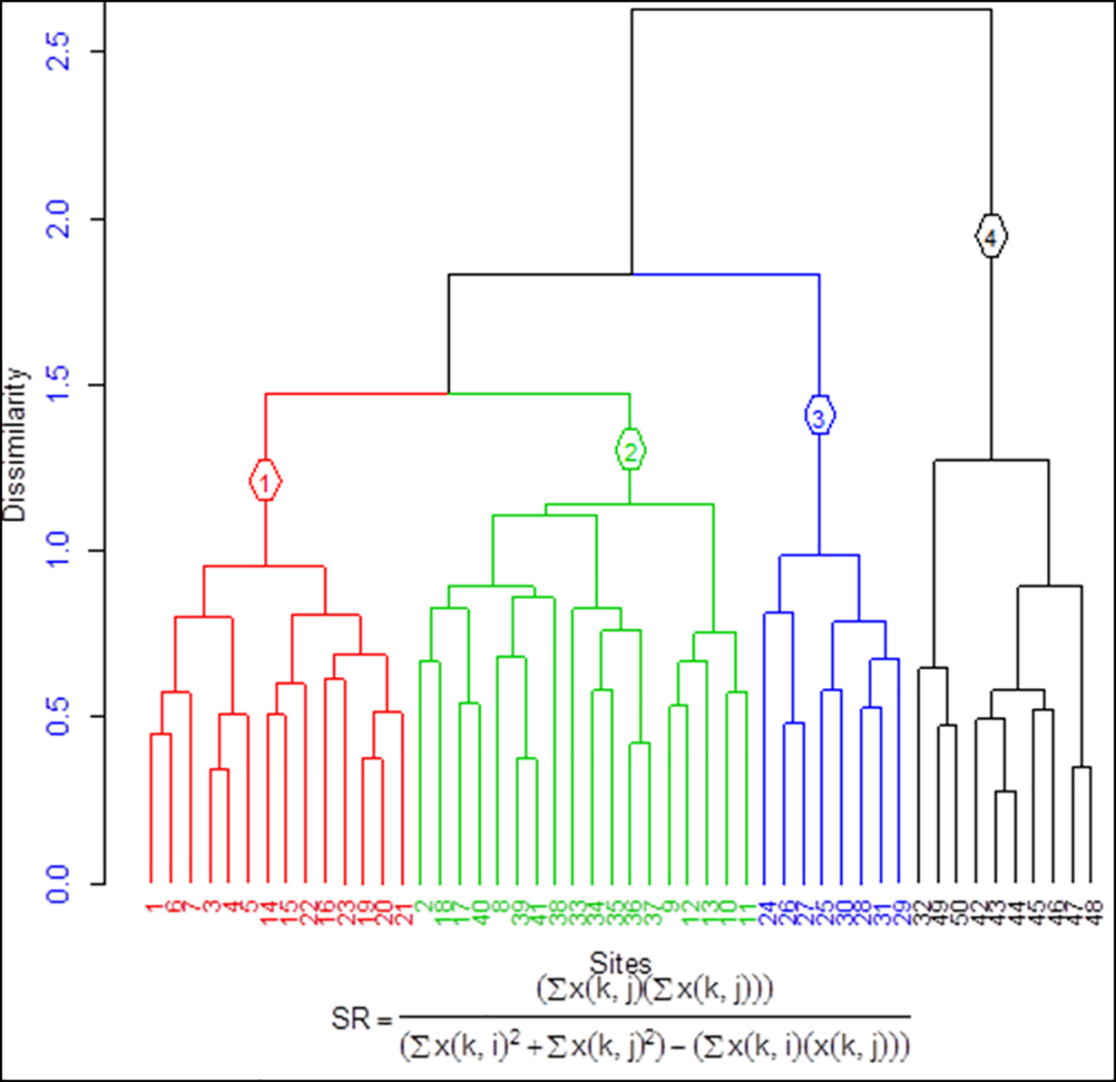
Dendrogram of hierarchical clustering using similarity ratio showing four community types in different sites (plot 1–50) of the study area (Ward's method, Euclidean distance). The vertical axis of the dendrogram represents the distance or dissimilarity between labeled clusters and the horizontal axis represents the sampled sitesclusteringinfour branches.

Four plant community types (clusters) weredeterminedat a vertical distancevalue of 1.25(between the default vertical distance values of 1.0 and 1.5) where the clusters are distinctly separate (Fig 2). The cluster of plot samples that grouped in one limb of tree is more similar in species composition and close proximity in altitude ranges than the other group of samples that are placed in other limbs of trees. The community types varied in size, ranging from 8–18 plots. The highest plot numbers were found in community type 2 (C2) whereas the least in C3. These two communities are rich in endemic taxa (Table 1). The community types were named by two characteristic species that have highest mean cover abundance value and/or both species were not found at least in one of the four clusters. Partial list ofcharacteristic species of each community types are given in S2 Appendix.

Community type 1: *Euclea divinorum*-*Maytenus arbutifolia* is represented by 14 plots that counted for 58 species. It is distributed in altitude ranges of 1986–2359 m a.s.l. Out of 58 species, three are restricted in this community type and the other 55 are commonly shared with other community types. Nearly half of the species are shared only with community type 2, which makes them more similar (Table 2). *Podocarpus falcatus* and *Syzygium guineense* are the dominant tree species. The three common species that exclusively found in this community type were: *Trichocladus ellipticus*, *Maytenus senegalensis*, and *Allophylus abyssinicus*.

**Table 2:** Similarity between the four plant community types ^a^(C1-C4) in Walga riparian vegetation.

	C1	C2	C3	C4
**C1**		CC*^b^ = 2x52/(17+19)	CC = 2x23/(45+16)	CC = 2x13/(55+19)
**C2**	2.89		CC = 2x23/(41+17)	CC = 2x15/(48+17)
**C3**	0.75	0.79		CC = 2x18/(21+14)
**C4**	0.35	0.46	1.03	

Note

^a^C1: *Euclea divinorum*-*Maytenus arbutifolia*, C2: *Pterolobium stellatum*- *Calpurnia aurea*, C3: *Brucea antidysenterica-Prunus africana*, C4: *Erica arborea-Hagenia abyssinica*.

*^b^Sorensen’s coefficient for community similarity. The formula is given in the upper-right and the calculated results are presented in the bottom-left sides of the table.

Community type 2: *Pterolobium stellatum*- *Calpurnia aurea* is distributed in the lowest altitude ranges between 1976–2212 m a.s.l. with some overlaps on community type 1. It is represented by highest number of plots (18) and species (66). Of the 66 species, 12 are restricted in this community type and 54 are commonly shared with other community types and largely with community type 1. *Ficus sur*, *Acacia abyssinca* and *Bersama abyssinica* Fresen. sub sp. abyssinica are the dominant species. There are 12 common plant species associated only with this community: *Ficus thonningii*, *Maytenus gracilipes*, *Senna singueana*, *Capparis tomentosa*, *Calpurnia aurea*, *Rytigynia neglecta*, *Allophyllus rubifolius*, *Grewia villosa*,*Ficus vasta*,*Ficus ovata*, *Opuntia ficus-indica* and *Acacia seyal*. This site is highly degraded by over grazing and expansion of agricultureexcept some inaccessible pocket areas.

Community type 3: *Brucea antidysenterica-Prunus africana* is located relatively in the mid altitude ranges between 2359-2676m a.s.l. It is represented by the lowest number of plots (8) with 48 species; out of which seven are restricted in this community type. The other 41 are commonly shared with other community types, largely with community type 4resulting in the second highest similarity coefficient (1.03) as shown in Table 2. *Bersama abyssinica*, *Erythrina brucei* and *Ficus sur* are the dominant canopy species. The seven common tree and shrub species associated only with this community type include: *Rhusglutinosa*, *Indigofera arrecta*, *Grewia velutina*, *Maytenus addat*, *Vernonia* sp., *Dodonaea angustifolia* and *Echinops longisetus*. It is relatively less degraded due to its steep slope topographical nature of the site and inaccessible for cattle grazing.

Community type 4: *Erica arborea-Hagenia abyssinica* is found at the highest altitude ranges between 2687–3078 m a.s.l. It is represented by 10 plots that comprised of 32 plant species; out of which 10 are exclusively found in this community type and the other 22 are commonly shared with other community types. *Erica arborea* and *Hagenia abyssinica* are the characteristic and dominant canopy species of this community. Other eight common tree and shrub species exclusively found in this community type include: *Myrsinemelanophloeos*, *Helichrysum argyranthum*, *Thymus schimperi*, *Pentas schimperiana*, *Protea gaguedi*, *Nuxia congesta*, *Rumex nervosus* and *Macowania abyssinica*.

The highest similarity is depicted between communitytypes 1 and 2 (CC = 2.89), followed by 3 and 4 while the least similarity is shown between communitytypes 1and 4with CC values of 0.35 (Table 2).

Shannon-Wiener diversity index was computed for the four plant community types of the Walga riparian vegetation (Table 3). The clusters are ranking in increasing order of total number of species in the community (richness) and diversity of 2> 1> 3 >4. Thus, community type 2 has the highest species diversity and richness whereas community type 4 has the least species diversity and richness. However, the highest value of Shannon’s equitability (evenness) is revealed in community type 3 though it has the smallest number of sampled quadrats (0.4 ha). This implies that relative abundance and even distribution of individuals of different species occurred in community type 3, followed by 2, 4 and 1.

**Table 3:** Species richness, evenness and diversity in the four plant community types.

Community Type	Species Richness (S)	Diversity index (H)	True diversity (D)	Shannon’s evenness (J)	H_max_
1 (18 plots)	58	3.60	37	0.89	4.06
2 (14 plots)	66	3.85	47	0.92	4.19
3 (8 plots)	48	3.58	36	0.93	3.87
4 (10 plots)	32	3.17	24	0.91	3.47
**Average**	**51**	**3.55**	**36**	**0.91**	**3.90**

**Fig 3:**
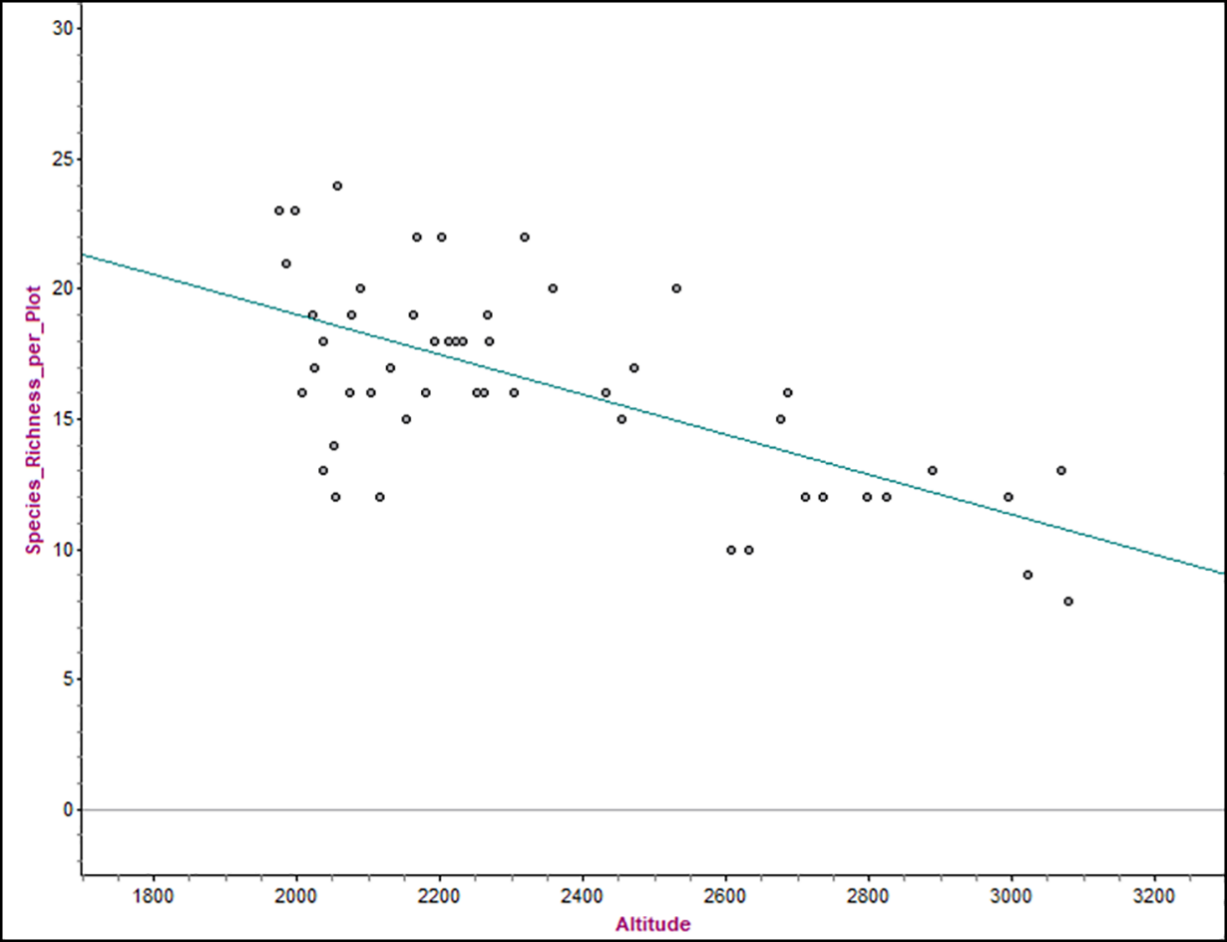
Scatter plots with least squares regression line showing relationship between patterns of species richness per plot and altitude. Equation of least square regression line:Species richness per plot = 34.387 − 0.00771558 Altitude (m a.s.l.). Correlation coefficient, r = -0.648, coefficient of determination, r^2^ = 0.420, and the best estimate for the slope is -0.00771558 +/-0.00262944 at a 95% of confidence level. The standard error of the regression slope is 0.0013, p<0.001.

The result shows a strong negative correlation (r = -0.65, p<0.001) between species richness per plot(1–50) and altitude gradient (from1976-3078 m a.s.l.)with 42% of the variation in species richnessbeing explained by altitude (Fig 3).The highest number of species (24) was recordedat plot two that was found in the lowest altitude ranges of community type 2 (1976–2212 m a.s.l.) while the least number of species (8) reported at plot 42 that was found at highest altitude ranges of community type 4 (2687–3078 m a.s.l.). Additionally, number of species in community types 1 (1986–2319 m a.s.l.) and 3(2359–2676 m a.s.l) fell approximately to nearby the regression line.

Community types1 and 2 were found in the downstream riparian vegetation (1976–2319 m a.s.l.), which had higher species (124) occurring in32 sampled quadrats (1.6 ha). There were 80 species occurring in 18 sampled quadrats (0.9 ha) in community types 3 and 4, at the upper stream (2359–3078 ma.s.l.). The upper stream riparian vegetation of Walga had higher number of species per ha (89) than the downstream riparian vegetation (76).

### The riparian vegetation structures in frequency, DBH and height class distributions of species

In the present study, *Carissa spinarum* is the most frequently observed species that occurred in 62% of the quadrats sampled, followed by *Bersama abyssinica* subsp. abyssinica and *Syzygium guineense* subsp. *guineense* in 60% of the quadrats. The remaining most frequently occurring species with more than 35% are given in Table 4.The frequency values of different species were classified into five frequency classes with nearly 15% interval each. A = 1–15, B = 16–30, C = 31–45, D = 46–62%. The result shows that 56% of the total woody species were distributed in the lowest frequency class (A), followed by 31%, 6% and 7% of the species in the frequency classes of B, C and D, respectively.

**Table 4:** Top 13 most frequently occurring riparian woody species out of fifty quadrats.

S. No.	Species	No. of Quadrats	Frequency (%)	Relative Frequency
1	*Carissa spinarum* L.	31	62	2.04
2	*Bersama abyssinica* Fresen. sub sp. abyssinica	30	60	2.38
3	*Syzygium guineense* (Willd.) DC. subsp. *guineense*	30	60	1.99
4	*Croton macrostachyus* Del.	26	52	1.84
5	*Rosa abyssinica* Lendley.	25	50	1.79
6	*Vernonia auriculifera* Hiern.	25	50	1.88
7	*Salix subserrata* Willd.	23	46	1.39
8	*Brucea antidysenterica* J. F. Mill.	22	44	1.86
9	*Ficus sur* Forssk.	22	44	1.81
10	*Maytenus arbutifolia* (A. Rich.) Wilczek var. arbutifolia	22	44	1.32
11	*Pterolobium stellatum* (Forssk.) Brenan	21	42	1.28
12	*Phoenix reclinata* Jacq.	20	40	1.21
13	*Myrica salicifolia* Hochst. ex A. Rich.	18	36	1.16

A total of 30 tree species were used for analyzing distribution of BA, stem density, DBH and height classes (S3 and S4 Appendices). A considerable numbers of individuals decreased with increasing in five DBH classes (they have already been specified in method section). In contrast to the percentage of total individuals, the distribution of BA of individuals of tree species increased with increasing DBH classes from 0.9–48.9%. The highest proportions of individuals are found in DBH classes 3 (27.9%) and 2 (25.4%) followed by 1(20.9%), 4 (17.5%) and 5 (8.3%). The total stem density (individuals of tree species/ha) with height ≥ 2.5 m was356 ha^-1^. Eight species including *Croton macrostachyus* (30.8 ha^-1^), *Syzygium guineense* subsp. *guineense* (28.8 ha^-1^), *Acacia abyssinica* (24.4 ha^-1^), *Podocarpus falatus* (24.0 ha^-1^), *Ficus sur* (22.0 ha^-1^) *Phoenix reclinata (18*.*0* ha^-1^*)*, *Myrica salicifolia (17*.*2* ha^-1^*) and Erytrina brucei* (16.8 ha^-1^) accounted for more than half of the total density (51.2%). The heights (m) of trees were distributed into five classes (Class 1 = 2.5–6.0, 2 = 6.1–12.0, 3 = 12.1–18.0, 4 = 18.1–24.0, 5 = ≥24.1). The highest proportion of density was recorded in height class 3 (30.6%), followed by height class 2 (26.7%), 1 (17.8%), 4 (16.7%) and 5 (8.0%). Generally, all the results depict that stem density increases with increasing percentage of total individual trees of different species in each height classes.

**Fig 4:**
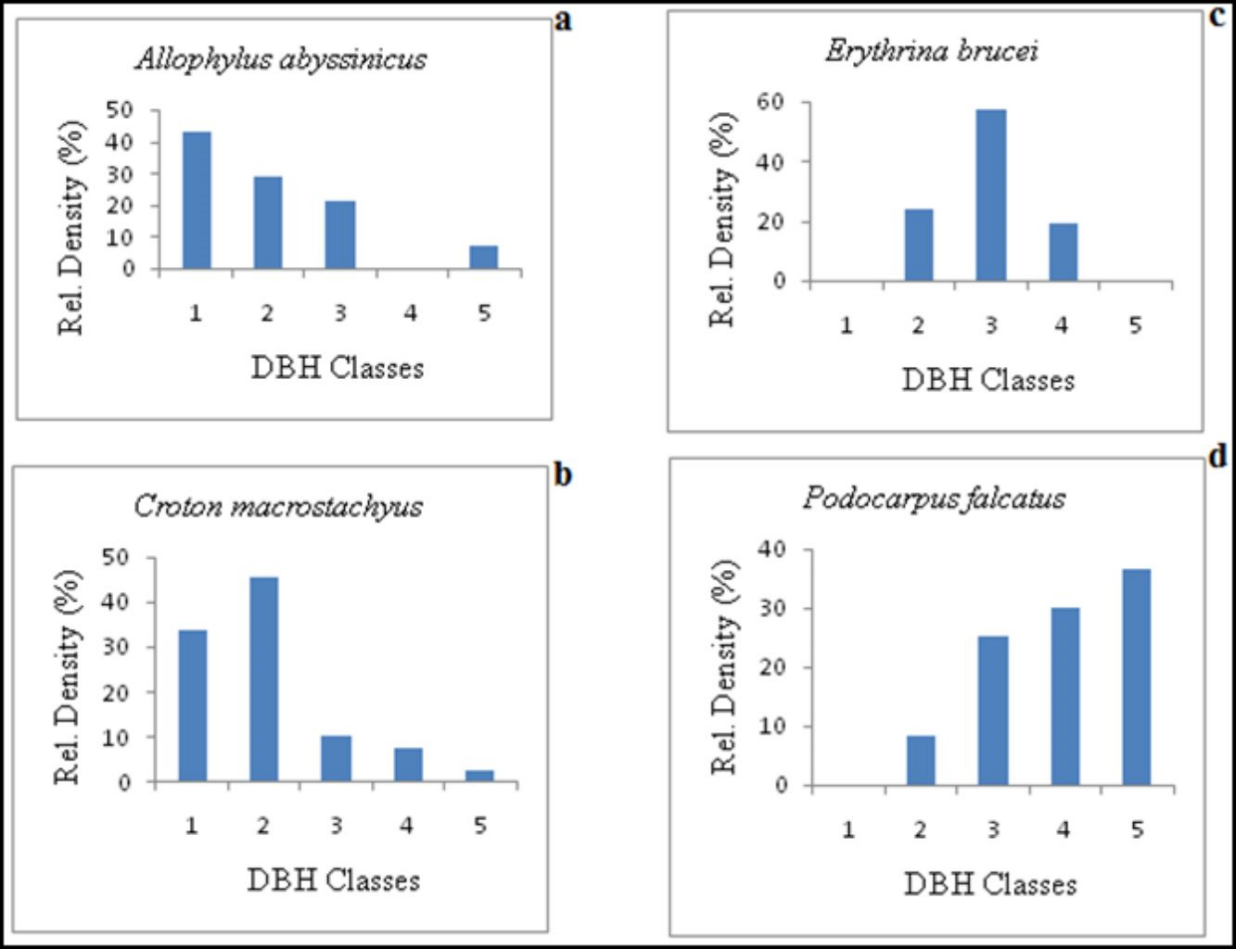
Four representative patterns of frequency distribution of tree density values against DBH classes in the riparian vegetation. Species represented by (a) *Allophylus abysinicus* (b) *Croton macrostachyus*, (c) *Erythrina brucei*, (d) *Podocarpus falcatus*. Class 1 = 2.5–10.0 cm, 2 = 10.1–20.0 cm, 3 = 20.1–30.0 cm, 4 = 30.1–40.0 cm, 5 = ≥ 40.1 cm.

The patterns of distribution of trees varied in each class. Fig 4A–4D shows four major distribution types of population patterns exemplified by *Allophylus abyssinicus*, *Croton macrostachyus*, *Erythrina brucei* and *Podocarpus falcatus*. The highest number of species was recorded in the first population structure.

The importance value index ranged from 1.5 to 13.0 in four community types. Community type 2 has relatively the highest values of IVI (26.7%), followed by 1 and 3. Only 14 woody species namely *Erythrina brucei*, *Ekebergia capensis*, *Croton macrostachyus*, *Brucea antidysenterica*, *Maesa lanceolata*, *Syzygium guineense* subsp. guineense, *Vernonia auriculifera*, *Ficus sur*, *Euclea divinorum*, *Juniperus procera*, *Rosa abyssinica*, *Discopodium penninervium*, *Ficus vasta* and *Olinia rochetiana* in that order accounted for one-fourth (27.0%) of the total IVI values (547.1).

### The relationship of environmental factors to the structure and distribution of riparian vegetation

**Fig 5:**
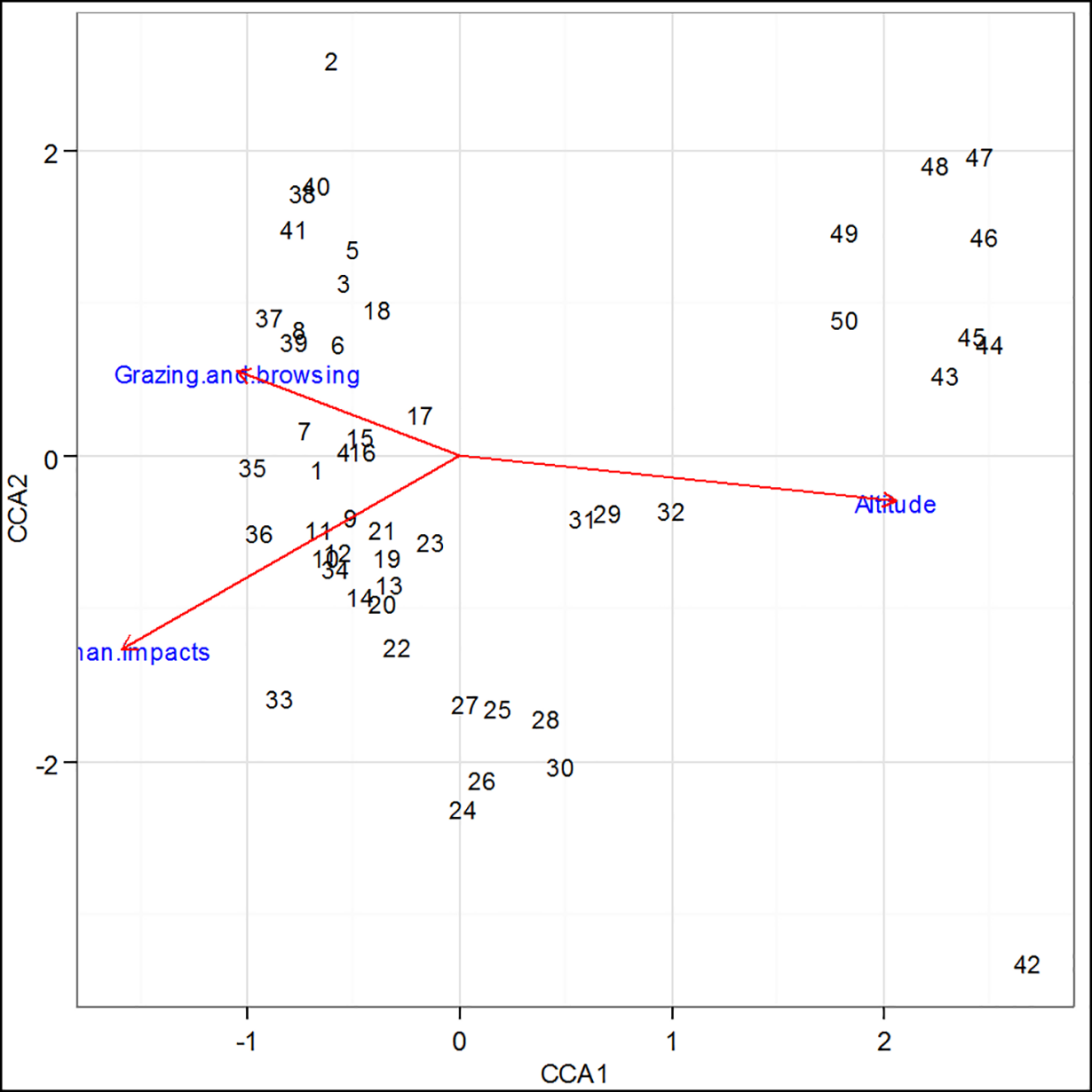
Canonical correspondence analysis diagram showing the ordination of community typesrepresented by plot numbers (1–50) and their correlation with environmental variables (altitude, grazing, human impacts). The direction and length of arrows shows the degree of correlation between plant community typesand the variables.

In this study, the environmental factors considered were altitude, grazing intensity and intensity of human impacts (S5 Appendix). Altitude showed strong positive relationships with community types 4 and 3. Human impacts were associated more with community type 2 in both axes (CCA2 and CCA1) while high intensity of grazing and browsing specifically occurred in community type 1 (Fig 5).

## Discussion

### Species composition, diversity and classification of plant communities

Walga riparian vegetation has relatively higher number ofwoody species (99) in comparison to other studies conducted elsewhere in Ethiopia [19, 20, 22] while it has less number of woody species (128) than Sire Beggo riparian vegetation [16]. Altitude and geographical location are major factors in the flora of riparian and swamp vegetation type [6] but edaphic factors, climatic conditions, tributaries, and the degree of human and livestock disturbances could also be considered responsible for the observed differences [35]. The Walga riparian vegetation varied in both species composition, diversity and even in growth forms of a single species with altitude gradients along the river at different microclimates. In contrast to other studies elsewhere in Ethiopia [19, 20], species richness per plot decreases with increasing altitude gradient.About 42%of the change in the patterns of species richness per plot wasassociatedwith the change in the altitude gradient (Fig 3).The remaining percentagescould be accounted for by the presence or absence of over livestock grazing and human oriented disturbances and other environmental factors.

The family Asteraceae came up with the highest number of species (12) in the study area. The second largest family was Fabaceae, which was represented by the highest number of species in other studies conducted in different parts of Ethiopia [16,19,20,22]. The study made in riparian vegetation elsewhere outside Ethiopia also showedthatFabaceae was the second richest family next to Moraceae [11]. Asteraceae and Fabaceae families have adapted more efficiently and successfully to seed dispersal mechanisms, primarily anemochory and zoochory that may make them the largest dicotyledonous families in the Flora with 440 and 620 species, respectively [6].

Thus, considering multiple environmental gradients would give a better picture of richness and the potential mechanisms responsible for distributing plant diversity in the riparian vegetation.The values of H range from 0.00 to 5.00, usually ranging from 1.50 to 3.50 [1,11,36]. Herein, the composite H average value of the entire riparian vegetation (3.55) is considered to be high.All community typesvary in number of species (richness), degree of dominance by one or a few species, relative abundance of all species (evenness) and number of growth forms. Magurran [36] and Jost et al. [34] confirmed that if a community has only a few species but much more in abundance, then species diversity is low. However, this is not the case for community 3 at the mid altitude ranges, which has the highest even distribution of individuals and species richness and diversity per area. Desalegn and Beierkuhnlein [21] reported that evenness and species richness at the mid altitude in southwest Ethiopian highlands was the result of a combination of climate-related water-energy dynamics, species-area relationships and local environmental factors, which have direct effects on plant physiological performance.

The Walgariparian floristic composition showed that the closest community typesin altitude ranges were getting more similarity in species composition than farthest ones. Forconformity ofdiversity of species differences between upper and downstream, several authors have also reported that the floristic composition of riparian vegetation is very variable and dependent on altitude and geographical location [16,19,20,22].

The phytogeographical similarity of Walga riparian vegetation with the four plant community types was compared with other riparian vegetation studied at different times in different parts of Ethiopia (Table 5). Table 5 indicates that Walga and Dabbis riparian vegetation score the highest species similarity index (0.52). Dabbis River is located nearby the present study area with altitude ranges of 2060–2204 m a.s.l. The highest species similarities could therefore be due to their geographical proximity and similar altitude ranges and agroecological zones. Conversely, poor species similarity is shared between Walga and Gilgel Gibe III riparian vegetation (0.16).Gilgel Gibe III is located in Omo River basin with altitude ranges of 680–1058 with 1426 mm and 17-29^0^c annual rainfall and temperature. Thus, the lowest species similarity may arise from the extreme variation in altitude ranges, climatic conditions, sample sizes (12 quadrats) and degree of anthropogenic impacts. The comparison made only for naturally growing tree and shrub species to keep its consistency.

**Table 5:** Comparison of the riparian vegetation of Walga with others studies in Ethiopia.

Riparian vegetation	Reference	S1^a^	S2^b^	2*C^c^	CC	Altitude range (m a.s.l.)	Location
**Dabbis**	[22]	70	42	58	0.52	2060–2204	Ambo District, Western Ethiopia
**Koga**	[19]	72	52	54	0.44	1894–2344	Merawi District, Northwestern Ethiopia
**Sire Beggo**	[16]	74	103	50	0.28	1247–1907	Gololcha District, Eastern Ethiopia
**Gilgel Gibe III**	[20]	86	73	26	0.16	680–1058	Mareka Gana District, Southwestern Ethiopia

Note: CC is Sorensen's coefficient for community similarity.

^a^ Number of species exclusively found in Walga.

^b^ number of species exclusively found in the other riparian vegetation that is in comparison with Walga.

^c^ for number of species common in both study areas.

### The riparian vegetation structure of species

The frequency distribution revealed that species in the lowest frequency class are recorded in few plots unlike to most frequent woody species which have good distribution status in the riparian vegetation (Table 4). In line with this, high percent of species in lower frequency classes points to a higher degree of floristic heterogeneity in the riparian vegetation [2,16]. Kent [2] also pointed out that the higher the frequency the more ecological important the plant is in the community.

In both DBH and height class distributions, higher percentage of species were recorded in the mid classes (2, 3, 4). On the other hand, the lowest percentage of species found in the lowest and highest classes (1 and 5) could be related to over grazing at young stage of plants and selective cutting of trees, respectively. In decreasing order of some species with percentage of BA ranges from 10.2 to 4%: *Ficus ovata*, *Ficus sur*, *Ficus vasta*, *Podocarpus falcatus*, *Syzygium guineense* subsp. guineense, *Euphorbia abyssinica*, *Juniperus procera*, *Ekebergia capensis*, *Allophylus abyssinicus*, *Croton macrostachyus*, *Erythrina brucei*, *Ficus thonningii*, *Hagenia abyssinica* and remnants are presented in S3 Appendix. These species are also listed in the top IVI and representative population structure patterns. Thus, species with the largest BA can be considered the most important woody species in the study area. A study on remnant afromontane forest on the central Platea of Shewa also confirmed that BA provides a better measure of the relative importance of the species than simple stem count [25]. Walga riparian vegetation has relatively higher importance value in comparison to other riparian vegetation in the southwestern Ethiopia such as, Gilgel Gibe III with 299.05 [20].

Population structure helps to examine the presence of a stable distribution that allows continuous regeneration [2]. The first population showed a pattern in which the density in DBH class one was the highest and decreased with increasing DBH class (Fig 4A). *Acacia seyal*, *Allophyllus abyssinicus*, *Bridelia micrantha*, *Celtis africana*, *Discopodium penninervium*, *Grewia trichocarpa*, *Maytenus addat*, *Phoenix reclinata*, and *Rhus longipes* showed this kind of distribution pattern. The second population pattern was characterized by the species having the highest density in the DBH class two and relatively higher density of individuals in the first DBH class and mainly decreasing successively towards the higher DBH classes with almost absence in some species in the 4^th^ and 5^th^ classes (Fig 4B). Seven tree species were marked to this population pattern: *Acacia abyssinica*, *Albizia schimperiana*, *Cassipourea malosana*, *Croton macrostachyus*, *Millettia ferruginea*, *Myrsine melanophloeos* and *Prunus africana*. The general pattern of the first and second DBH classes' distribution of the woody species showed an inverted J-shape with many small stems compared to few large ones. Such reversed J-shaped distribution pattern depicts that naturally the area is on the status of healthy regeneration and recruitment potential. However, it is not free from selective cutting of trees for various purposes.

The third population pattern was represented by species having the highest density in the middle DBH class, and lower values at both ends (Fig 4C). *Erythrina brucei*, *Euphorbia abyssinica*, *Ficus thonningii*, *Hagenia abyssinica*, *Myrica salicifolia*, *Nuxia congesta* and *Olea europaea* subsp. cuspidata showed this kind of distribution pattern. This kind of distribution pattern can be attributed to selective cutting of trees, over grazing and browsing at young stages for some species and slow germination rate due to stony endocarp (e.g., *O*.*europaea* subsp. cuspidata). This ‘bell-shaped’ type distribution had also been reported by Bekele [25]. The forth population pattern was represented by the species which had the highest density in DBH classes five or four and then decreasing towards the lowest DBH class (Fig 4D). This population pattern includes: *Ekebergia capensis*, *Ficus ovata*, *Ficus sur*, *Ficusvasta*,*Juniperus procera*,*Podocarpus falcatus* and *Syzygium guineense* subsp. *guineense*. This is the J-shaped pattern of species population structure in which reproduction (Seed germination) of the species is poor, particularly in gymnosperm groups such as, *Juniperus procera* and *Podocarpus falcatus* [16,25]. Anotherreason for small number of individuals in lower diameter classes than higher diameter classes couldbe due to selective cutting of trees at lower and middle classes.

### Impacts of environmental variables on plant distribution in different communities

Manyworks have shown that disturbance of any kind could have a large impact on species diversity and abundance in plant community [20,37,38]. The Walga riparian plant communities experience two main types of disturbances: natural and anthropogenic. Natural factors in riparian ecosystems include a canopy gap creation due to invasive species, flooding and species restriction within a certain altitude ranges. Anthropogenic disturbances include construction of dams, clearing forests for agriculture, *Eucalyptus* plantation, selective cutting trees and excessive livestock grazing.

Ordination using CCA reflected that altitude had a remarkable effect on distribution of plant species along the river bank (Fig 5). In some studies, highest species richness and diversity were recorded at high altitude [19, 20] and vice versa [16] while other study did not show much strong response to altitude [21]. The anthropogenic impacts are more or less associated with the whole vegetation but the level of intensity is clearly marked in community types1and 2because the slope and preferable plant materials of those community types was appropriate for those activities. Removals of plant cover by extensive livestock grazing and expansion of agriculture are usually identified as the major threats in different parts of Ethiopia [1,25, 27].

### Endemic species and conservation status of the riparian vegetation

According to Vivero et al. [26] and the IUCN Red List Criteria [39], three species listed wereunder the category of near threatened (*Crotalaria rosenii*, *Inula confertiflora* and *Maytenus addat*) and the other eight were found under the category of least concern plant species (Table 1). This implies to three of them are close to qualifying for, or are likely to qualify for, a threatened category in the near future. Hence, special attention is needed forthose that under near threatened species. This can be achieved through holistic conservation of the riparian ecosystem with special priority to most affected community types.

Probably one of the most important roles of riparian vegetation is buffering between terrestrial and aquatic systems, which in turn keeps its ecological, economic and social benefits of the riparian zone. With removal of the vegetation cover or the impoverishment of the biodiversity all the ecosystem goods and services will be lost.Wise management of riparian vegetation is thus required in the study area. According to Gemeda et al. [22], the major mitigation measures suggested by the local community in decreasing percentage frequency include creating awareness, control livestock grazing and enclosure of riparian vegetation through various means. It is obvious that protection of riparian vegetation was not only left to governmental bodies rather it should be everyone of concern. A combination of conservation with rehabilitation and restoration in mind is vital for maintaining the riparian vegetation. The details of some of these rehabilitation techniques (such as re-plantation with indigenous plant species, reducing grazing pressure) and strategies of restoration (both active and passive) were proposed to be effective in keep up the sustainable conservation of ecosystems[4,7,15]. Thus, reducing grazing pressures through fencing, removal of selective cutting of trees and expansion of agriculture are some of the techniques which are vital to recover natural riparian vegetation in the present study area. However, the application of all these rehabilitation techniques and passive restoration strategies throughout the entire Walga riparian system may cost a lot. Therefore, specific priorities should be given for plant community types2 and 3, which are more known in species richness and endemism than those of plant community types1 and 4.

## Conclusions

The present study describes the riparian floristic communities along an altitude gradient on the Walga River in the southwestern Ethiopia. These floristic composition groups are characterized by species richness, diversity, and evenness matrices and correlated with livestock grazing and browsing, human disturbance, and altitude. Additionally, forest structure measurements were collected on a subset of riparian trees and analyzed for population structure. The result shows that about 42% of the variation in species richness per plot being explained by altitudegradient. Despite theirsmaller numbers of sampled quadrat, the upper stream riparian vegetation of Walga(2359–3078 m a.s.l.) had higher number of species per ha (89) than the downstream riparian vegetation (76). Human and livestock activities are correlated both with specific community typesand altitude because altitude is correlated with all community types while it is more strongly correlated with community types 3 and 4. Thus, considering multiple environmental gradients would give a better picture of richness and the potential mechanisms responsible for distributing plant diversity in the riparian vegetation.Although it is widely interrupted by human and livestock disturbances, Walga riparian vegetation is remain rich in plant diversity (99 species) with more than 10% of them are endemic to the Flora area. Based on our finding, the highest species diversity, richness, endemism and even distribution of individuals occurred in community types2 (*Pterolobium stellatum*—*Calpurnia aurea* in the lowest altitude ranges between 1976–2212 m a.s.l.) and 3 (*Brucea antidysenterica*-*Prunus africana* in the mid altitude ranges between 2359–2676 m a.s.l.). Therefore, these sites need special priority to conserve majority of species which are ecologically very important and harbor nine endemic taxa. In conclusion, the finding of this paper provides information that is necessary for making conservation and management practices.

## Supporting information

S1 Appendix Inventory of plant species collected in Walga riparian vegetation. Note: ^+^Stands for endemic Species and ++ refers to near endemic species that are found only in Ethiopia and Eritrea, Missing plot numbers are those which have no new species encountered other than species already recorded in the preceding plots.
(PDF)

S2 Appendix Synoptic table of characteristic and dominant species of the study area. The occurrences of non-characteristic species with % values < 2.00 are not shown. Some of the species common but notably less characteristics are not listed here.
(PDF)

S3 Appendix Distribution of basal area of tree species in the DBH classes. (PDF)

S4 Appendix Stem density and height classes of tree species of the study area. (PDF)

S5 Appendix Environmental factors associated with Walga Riparian Vegetation and location of plots. (PDF)
