# Supplementary material for: Woody species composition and diversity of riparian vegetation along the Walga River, Southwestern Ethiopia
Source: PLoS One. 2018 Oct 17;13(10):e0204733. doi: 10.1371/journal.pone.0204733 (PMC6192589; doi:10.1371/journal.pone.0204733)
Supplement: S1 Appendix — Note: +Stands for endemic Species and ++ refers to near endemic species that are found only in Ethiopia and Eritrea, Missing plot numbers are those which have no new species encountered other than species already recorded in the preceding plots. (PDF) [file pone.0204733.s001.pdf]

No.	**Scientific name**	**Local name (Oromic/Amharic)**	**Family**	**Ha**	**Geographical location (based on GPS record)**	**Plot No.**	**Collection No.**
1	*Acacia abyssinica* Hochst.	Laaftoo / Girar	Fabaceae	T	2055 m; 08^{0}34.515'; 037^{0}56.862'	1	MM363
2	*Acacia seyal* Del.	Girar Wacho	Fabaceae	T	1998 m; 08^{0}32.459'; 037^{0}55.934'	39	MM440
3	*Agarista salicifolia* (Comm. ex Lam.) Hook.	Sootirii/Ketem	Ericaceae	S/T	2798 m; 08^{0}45. 915'; 037^{0}53.481'	48	MM456
4	Agave sisalana Perro ex Eng.	Algae/Chiret	Agavaceae	S	2088 m; 08^{0}35.643'; 037^{0}57.220'	5	MM401
5	*Albizia schimperiana* Oliv.	Mukaarbaa/Sesa	Fabaceae	T	2057 m; 08^{0}34.830'; 037^{0}56.981'	2	MM393
6	*Allophyllus rubifolius* (Hochst ex A.Rich.)Engl.	Urdufarda/Embis	Sapindaceae	S	2057 m; 08^{0}34.830'; 037^{0}56.981'	2	MM383
7	*Allophylus abyssinicus* (Hochst.)	Sarara/Embis	Sapindaceae	T	2269 m; 08^{0}40.224'; 037^{0}56.313'	21	MM420
8	*Asparagus africanus* Lam.	Seertii/Qestanicha	Asparagaceae	S	2057 m; 08^{0}34.830'; 037^{0}56.981'	2	MM391
9	*Bersama abyssinica* Fresen. sub sp. abyssinica	Lolchiisaa /Azamr	Melianthaceae	S/T	2055 m; 08^{0}34.515'; 037^{0}56.862'	1	MM367
10	*Bridelia micrantha* (Hochst.) Baill.	Yenebir tifir	Euphorbiaceae	T	2052 m; 08^{0}34.183'; 037^{0}56.616'	33	MM454
11	*Brucea antidysenterica* J. F. Mill.	Qomengo /Waginos	Simaroubaceae	S/T	2055 m; 08^{0}34.515'; 037^{0}56.862'	1	MM365
12	*Buddleja polystachya* Fresen.	Qawwisa/Amfar	Loganiaceae	S/T	2359 m; 08^{0}41.316'; 037^{0}55.450'	24	MM421
13	*Calpurnia aurea* (Ait.) Benth.	Ceekaa /Digta	Fabaceae	S	2037 m; 08^{0}33.958'; 037^{0}56.455'	34	MM438
14	*Capparis tomentosa* Lam.	Guracha /Gimero	Capparidaceae	S	2037 m; 08^{0}33.958'; 037^{0}56.455'	34	MM439
15	*Carissa spinarum* L.	Agamsa/Agam	Apocynaceae	S	2055 m; 08^{0}34.515'; 037^{0}56.862'	1	MM362
16	*Cassipourea malosana* (Baker) Alston	Galisaa/Buna qtel	Rhizophoraceae	T	2057 m; 08^{0}34.830'; 037^{0}56.981'	2	MM381
17	*Celtis africana* Burm. f	Ameleqa	Ulmaceae	T	2359 m; 08^{0}41.316'; 037^{0}55.450'	24	MM419
18	*Clausena anisata* (Willd.) Benth.	Ulmaayii /Limich	Rutaceae	S/T	2055 m; 08^{0}34.515'; 037^{0}56.862'	1	MM366
19	*Clerodendrum myricoides* (Hochst.) Vatke	Marasisaa/Misirich	Lamiaceae	S	2088 m; 08^{0}35.643'; 037^{0}57.220'	5	MM403
20	*Conyza hypoleuca* A. Rich.	Balado/Nechillo	Asteraceae	S	2687 m; 08^{0}44.671'; 037^{0}53.759'	32	MM435
21	*Crotalaria rosenii*+ (Pax.) Milne-Redh. ex	Abachonanie Enchet	Fabaceae	S	2075 m; 08^{0}35.360'; 037^{0}56.116'	4	MM398

	Polhill						
22	*Croton macrostachyus* Del.	Bakkanniissa/Bissana	Euphorbiaceae	T	2055 m; 08^{0}34.515'; 037^{0}56.862'	1	MM368
23	*Dalbergia lactea* Vatke	---------	Fabaceae	S	2057 m; 08^{0}34.830'; 037^{0}56.981'	2	MM374
24	*Discopodium penninervium* Hochst.	Chochinga/Qelaho	Solanaceae	T	2319 m; 08^{0}41.037'; 037^{0}55.787'	23	MM418
25	*Dodonaea angustifolia* L. f.	Ittacha/Kitkita	Sapindaceae	S	2359 m; 08^{0}41.316'; 037^{0}55.450'	24	MM427
26	*Dovyalis abyssinica* (A. Rich.) Warb.	Akuukkuu/Koshm	Flacourtiaceae	S	2267 m; 08^{0}39.891'; 037^{0}56.437'	20	MM451
27	*Echinops longisetus*+ A. Rich.	Sokorru/ Keylo	Asteraceae	S	2359 m; 08^{0}41.316'; 037^{0}55.450'	24	MM424
28	*Echinops pappii* Chiov.	Barwerante/Kushele	Asteraceae	S	2057 m; 08^{0}34.830'; 037^{0}56.981'	2	MM378
29	*Ekebergia capensis* Sparrm.	Somboo/Lol	Meliaceae	T	2103 m; 08^{0}35.913'; 037^{0}57.252'	6	MM405
30	*Embelia schimperi* Vatke	Anku/Enqoqo	Myrsinaceae	S/T	2472 m; 08^{0}43.271'; 037^{0}54.453'	27	MM422
31	*Erica arborea* L.	Kemexii/Asta	Ericaceae	S/T	3078 m; 08^{0}47.122'; 037^{0}51.796'	42	MM441
32	*Erythrina brucei* +Schweinf.	Waleensuu/Quara	Fabaceae	T	2163 m; 08^{0}37.186'; 037^{0}57.162'	10	MM423
33	*Euclea divinorum* Hiern	Mi'eessaa/Dedeho	Ebenaceae	S	2057 m; 08^{0}34.830'; 037^{0}56.981'	2	MM389
34	*Euclea racemosa* Murr. Subsp. Schimperi	Mieessaa/Dedeho	Ebenaceae	S/T	2075 m; 08^{0}35.360'; 037^{0}56.116'	4	MM396
35	*Euphorbia abyssinica* Gmel.	Adaamii/Qulqual	Euphorbiaceae	T	2088 m; 08^{0}35.643'; 037^{0}57.220'	5	MM400
36	*Ficus ovata* Vahl	Dembi/Shoola	Moraceae	T	1976 m; 08^{0}31.752'; 037^{0}55.894'	41	MM397
37	*Ficus sur* Forssk.	Harbu	Moraceae	T	2261 m; 08^{0}39.650'; 037^{0}56.639'	19	MM447
38	*Ficus thonningii* Blume	Dembi/Chbaha	Moraceae	T	2168 m; 08^{0}37.469'; 037^{0}57.076'	11	MM413
39	*Ficus vasta* Forssk.	Qilxuu /Warka	Moraceae	T	2057 m; 08^{0}34.830'; 037^{0}56.981'	2	MM382
40	*Flacourtia indica* (Burm.f.) Merr.	Akuukkuu/Qurqura	Flacourtiaceae	S/T	2057 m; 08^{0}34.830'; 037^{0}56.981'	2	MM380
41	*Grewia ferruginea* Hochst.ex A. Rich.	Lenquata	Tiliaceae	S	2024 m; 08^{0}33.655'; 037^{0}56.409'	35	MM447
42	*Grewia trichocarpa* Hochst. ex A. Rich.	Araarasaa	Tiliaceae	T	2057 m; 08^{0}34.830'; 037^{0}56.981'	2	MM390
43	*Grewia velutina* (Forssk.) Vahl.	Dhoqonu/Lenquata	Tiliaceae	S/T	2531 m; 08^{0}43.614'; 037^{0}54.306'	28	MM458
44	*Grewia villosa* Willd.	Dhoqonu/Agobday	Tiliaceae	S	2057 m; 08^{0}34.830'; 037^{0}56.981'	2	MM379
45	*Hagenia abyssinica* (Bruce) J.F.Gmelin	Hexxoo/Kosso	Rosaceae	T	3022 m; 08^{0}46.980'; 037^{0}52.053'	44	MM446

46	*Helichrysum argyranthum* O.Hoffm.	Necho	Asteraceae	S	2888 m; 08^{0}46.654'; 037^{0}52.918'	46	MM449
47	*Hypericum revolutum* Vahl	Amja	Hypericaceae	S/T	2057 m; 08^{0}34.830'; 037^{0}56.981'	2	MM392
48	*Indigofera arrecta* Hochst. ex A. Rich. Gillett	Birbiraa /Dgndg	Fabaceae	S	2455 m; 08^{0}43.068'; 037^{0}54.594'	26	MM450
49	*Inula confertiflora*$^+$ A. Rich.	Bulaancoo /Weynagift	Asteraceae	S	2633 m; 08^{0}43.749'; 037^{0}54.011'	29	MM433
50	*Juniperus procera* Hochst. ex. Endl.	Gaattiraa habasha	Cupressaceae	T	2181 m; 08^{0}37.732'; 037^{0}56.987'	12	MM414
51	*Laggera tomentosa+* (Sch. Bip.ex A. Rich.) Oliv. & Hiern.	Kaskasse/Alashume	Asteraceae	S	2433 m; 08^{0}42.683'; 037^{0}54.780'	25	MM430
52	*Lippia adoensis* Hochst. ex Walp. var adoensis++	Kusaayee/ Koseret	Verbenaceae	S	2154 m; 08^{0}36.930'; 037^{0}57.214'	9	MM411
53	*Lobelia giberroa* Hemsl.	Daju/Jibera	Lobeliaceae	S	2687 m; 08^{0}44.671'; 037^{0}53.759'	32	MM431
54	*Macowania abyssinica* (Sch.Bip.ex Walp.) B.L.Burtt	Adado/Necho	Asteraceae	S	2888 m; 08^{0}46.654'; 037^{0}52.918'	46	MM444
55	*Maesa lanceolata* Forssk.	Abbayyii/Qelewa	Myrsinaceae	S/T	2076 m; 08^{0}35.111'; 037^{0}56.040'	3	MM395
56	*Maytenus addat+* (Loes.) Sebsebe	Kombolcha/Atatt	Celastraceae	T	2359 m; 08^{0}41.316'; 037^{0}55.450'	24	MM428
57	*Maytenus arbutifolia* (A. Rich.) Wilczek var. arbutifolia	Kombolcha /Atatt	Celactraceae	S/T	2055 m; 08^{0}34.515'; 037^{0}56.862'	1	MM361
58	*Maytenus gracilipes* (Welw. Ex Oliv.) Excell	Kombolcha/Atatt	Celastraceae	S	2057 m; 08^{0}34.830'; 037^{0}56.981'	2	MM385
59	*Maytenus senegalensis* (Lam.) Exell	Firkuta/Atatt	Celastraceae	S	2192 m; 08^{0}38.265'; 037^{0}56.983'	14	MM425
60	*Microglossa pyrifolia* (Lam.) *Kuntze*	Entesa	Asteraceae	S	2088 m; 08^{0}35.643'; 037^{0}57.220'	5	MM402
61	*Millettia ferruginea+* (Hochst.) Bark.	Sootaloo/Birbira	Fabaceae	T	2057 m; 08^{0}34.830'; 037^{0}56.981'	2	MM375
62	*Myrica salicifolia* Hochst. ex A. Rich.	Borodo/Shinet	Myricaceae	T	2075 m; 08^{0}35.360'; 037^{0}56.116'	4	MM399
63	*Myrsine africana* L.	Qacama/Kechemo	Myrsinaceae	S	2057 m; 08^{0}34.830'; 037^{0}56.981'	2	MM384
64	*Myrsine melanophloeos* (L.) R. Br.	Weyil	Myrsinaceae	T	2824 m; 08^{0}46. 553'; 037^{0}53.617'	47	MM443

65	*Nuxia congesta* R.Br. ex Fresen.	Qawwisa/Askwar	Loganiaceae	T	2996 m; 08^{0}46.848'; 037^{0}52.529'	45	MM448
66	*Ocimum lamiifolium* Hochst.ex Benth.	Ancabbii/Damakessie	Lamiaceae	S	2057 m; 08^{0}34.830'; 037^{0}56.981'	2	MM387
67	*Olea europaea* L. subsp. cuspidata	Ejersa/Weyira	Oleaceae	T	2251 m; 08^{0}39.460'; 037^{0}56.926'	18	MM452
68	*Olinia rochetiana* A. Juss	Daalachoo/Asqamo	Oliniaceae	S/T	2433 m; 08^{0}42.683'; 037^{0}54.780'	25	MM429
69	*Opuntia ficus-indica* (L.) Miller.	Adaamii/Shele qulqual	Cactaceae	S	2008 m; 08^{0}32.769'; 037^{0}56.050'	38	MM360
70	*Osyris quadripartita* Decn.	Waatoo/Keret	Santalaceae	S	2088 m; 08^{0}35.643'; 037^{0}57.220'	5	MM404
71	*Pavetta oliveriana* Hiern	Qaqessaa/Kamadua	Rubiaceae	S	2057 m; 08^{0}34.830'; 037^{0}56.981'	2	MM377
72	*Pentas schimperiana* (A. Rich.) Vatke	Qaasii/Weynagift	Rubiaceae	S	3078 m; 08^{0}47.122'; 037^{0}51.796'	42	MM442
73	*Phoenix reclinata* Jacq.	Meexii/Zenbaba	Arecaceae	T	2055 m; 08^{0}34.515'; 037^{0}56.862'	1	MM369
74	*Podocarpus falcatus* (Thunb.) Mirb.	Birbirisa/Zigba	Podocarpaceae	T	2055 m; 08^{0}34.515'; 037^{0}56.862'	1	MM370
75	*Premna schimperi* Engl.	Urgeessaa	Lamiaceae	S	2130 m; 08^{0}36.407'; 037^{0}57.197'	8	MM410
76	*Protea gaguedi* J. F. Gmel.	Daanisa/Yahya zeng	Proteaceae	S	2735 m; 08^{0}45. 457'; 037^{0}53.606'	49	MM432
77	*Prunus africana* (Hook. f.) Kalkm.	Hoomii/Tiqur Inchet	Rosaceae	T	222 m; 08^{0}38.865'; 037^{0}57.027'	16	MM417
78	*Pterolobium stellatum* (Forssk.) Brenan	Harangamaa /Qontr	Fabaceae	S	2057 m; 08^{0}34.830'; 037^{0}56.981'	2	MM373
79	*Rhus glutinosa* A. Rich. *Subsp. neoglutinosa+*	Dabobesa/Embis	Anacardiaceae	S/T	2359 m; 08^{0}41.316'; 037^{0}55.450'	24	MM426
80	*Rhus longipes* Engl.	Tatessaa	Anacardiaceae	T	2076 m; 08^{0}35.111'; 037^{0}56.040'	3	MM394
81	*Rhus vulgaris* Meikle	Dabobesa/Embis	Anacardiaceae	S/T	2203 m; 08^{0}38.556'; 037^{0}57.004'	15	MM416
82	*Ricinus communis* L.	Qobboo/Gulo	Euphorbiaceae	S	2163 m; 08^{0}37.186'; 037^{0}57.162'	10	MM412
83	*Ritchiea albersii Gilg*	Avokar/Dyngay seber	Capparidaceae	S	2055 m; 08^{0}34.515'; 037^{0}56.862'	1	MM372
84	*Rosa abyssinica* Lendley.	Qaqawwee /Kega	Rosaceae	S	2057 m; 08^{0}34.830'; 037^{0}56.981'	2	MM386
85	*Rubus apetalus* Poir	Goraa/ Enjorie	Rosaceae	S	2057 m; 08^{0}34.830'; 037^{0}56.981'	2	MM376
86	*Rumex nervosus* Vahl.	Dhangaggoo/Embuacho	Polygonaceae	S	2735 m; 08^{0}45. 457'; 037^{0}53.606'	49	MM436
87	*Rytigynia neglecta (Hierns) Robyns*	Mixo	Rubiaceae	S	1986 m; 08^{0}32.076'; 037^{0}55.855'	40	MM453
88	*Salix subserrata* Willd.	Alaltuu/Riga	Salicaceae	S	2057 m; 08^{0}34.830'; 037^{0}56.981'	2	MM388

89	*Senna singueana (Del.) Lock*	Gufa	Fabaceae	S	2052 m; 08^{0}34.183'; 037^{0}56.616'	33	MM437
90	*Sida schimperiana* Hochst. ex A. Rich.	Mila harree/Chifrig	Malvaceae	S	2117 m; 08^{0}36.130'; 037^{0}57.200'	7	MM407
91	*Solanecio gigas*+ (Vatke) C. Jeffrey	Osole/shikok gomen	Asteraceae	S	2608 m; 08^{0}44.118'; 037^{0}53.918'	30	MM434
92	*Solanum incanum* L.	Hiddi/Yedi	Solanaceae	S	2130 m; 08^{0}36.407'; 037^{0}57.197'	8	MM409
93	*Syzygium guineense* (Willd.) DC. subsp. *guineense*	Goosuu/Doqma	Myrtaceae	T	2055 m; 08^{0}34.515'; 037^{0}56.862'	1	MM371
94	*Thymus schimperi* ++ Ron. subsp. schimperi	Tosign	Lamiaceae	S	3022 m; 08^{0}46.980'; 037^{0}52.053'	44	MM445
95	*Trichocladus ellipticus* EckL. & Zeyh. subsp. malosanus	Madessa/Adesa	Hamamelidaceae	S/T	2103 m; 08^{0}35.913'; 037^{0}57.252'	6	MM406
96	*Triumfetta brachyceras* K. Schum.	Danigola/Nacha	Tiliaceae	S	2117 m; 08^{0}36.130'; 037^{0}57.200'	7	MM408
97	*Vernonia amygdalina* Del.	Eebicha/Grawa	Asteraceae	S	2212 m; 08^{0}38.002'; 037^{0}56.908'	13	MM415
98	*Vernonia auriculifera* Hiern.	Reejii /Gujo	Asteraceae	S	2055 m; 08^{0}34.515'; 037^{0}56.862'	1	MM364
99	*Vernonia* sp.	-------	Asteraceae	S	2676 m; 08^{0}44.485'; 037^{0}53.874'	31	MM455
