# Supplementary material for: Woody species composition and diversity of riparian vegetation along the Walga River, Southwestern Ethiopia
Source: PLoS One. 2018 Oct 17;13(10):e0204733. doi: 10.1371/journal.pone.0204733 (PMC6192589; doi:10.1371/journal.pone.0204733)
Supplement: S2 Appendix — The occurrences of non-characteristic species with % values < 2.00 are not shown. Some of the species common but notably less characteristics are not listed here. (PDF) [file pone.0204733.s002.pdf]

Species	C 1	C 2	C 3	C 4
Euclea divinorum	**2.00**	0.83	0.38	0.00
Syzygium guineense subsp. guineense	3.71	2.11	3.38	0.40
Maytenus arbutifolia var. arbutifolia	**2.36**	1.17	0.50	0.00
Croton macrostachyus	2.07	2.17	1.12	0.00
Carissa spinarum	2.36	1.50	2.38	0.20
Pterolobium stellatum	1.79	**2.00**	0.61	0.00
Calpurnia aurea	0.00	**1.50**	0.00	0.00
Salix subserrata	2.21	0.72	2.75	0.30
Ficus sur	1.14	2.56	1.25	0.00
Bersama abyssinica sub sp. abyssinica	2.00	2.56	1.38	0.00
Brucea antidysenterica	1.57	0.00	**2.17**	0.30
Prunus africana	0.21	0.00	**1.33**	0.00
Acacia abyssinica	0.93	0.33	3.00	0.40
Olinia rochetiana	0.00	0.33	0.38	2.10
Conyza hypoleuca	0.00	0.22	0.00	2.10
Erica arborea	0.00	0.00	0.00	**5.20**
Hagenia abyssinica	0.00	0.00	0.00	**2.30**
