# Supplementary material for: Woody species composition and diversity of riparian vegetation along the Walga River, Southwestern Ethiopia
Source: PLoS One. 2018 Oct 17;13(10):e0204733. doi: 10.1371/journal.pone.0204733 (PMC6192589; doi:10.1371/journal.pone.0204733)
Supplement: S3 Appendix — (PDF) [file pone.0204733.s003.pdf]

Name of Species	DBH Classes (cm)					Average DBH in cm	Basal Area of tree sp.	% of Basal Area
	2.5-10.0	10.1-20.0	20.1-30.0	30.1-40.0	≥ 40.1			
Acacia abyssinica Hochst.	6	30	20	5	0	20.5	0.013	2.7
Acacia seyal Del.	6	3	0	0	0	10.8	0.004	0.7
Albizia schimperiana Oliv.	12	15	8	3	0	20.5	0.013	2.7
Allophylus abyssinicus (Hochst.)	12	8	6		2	25.4	0.020	4.1
Bridelia micrantha (Hochst.) Baill.	4	3	2			15.6	0.008	1.5
Cassipourea malosana (Baker) Alston	0	6	2		0	20	0.013	2.5
Celtis africana Burm. f.	12	6	5	0	0	15.6	0.008	1.5
Croton macrostachyus Del.	26	35	8	6	2	25.4	0.020	4.1
Discopodium penninervium Hochst.	14	3	0	0	0	10.8	0.004	0.7
Ekebergia capensis Sparrm.	0	2	6	0	10	26.3	0.022	4.4
Erythrina brucei Schweinf.	0	10	24	8	0	25	0.020	4.0
Euphorbia abyssinica Gmel.	0	0	16	6	0	30	0.028	5.7
Ficus ovata Vahl	0	0	0	7	5	40	0.050	10.2
Ficus sur Forssk.	0	0	20	27	8	36.8	0.043	8.6
Ficus thonningii Blume	0	0	7	0	0	25	0.020	4.0
Ficus vasta Forssk.	0	0	0	11	5	35	0.038	7.8
Grewia trichocarpa Hochst. ex A. Rich.	16	4	0	0	0	10.8	0.004	0.7
Hagenia abyssinica (Bruce) J.F.Gmelin	0	2	10	5	0	25	0.020	4.0
Juniperus procera Hochst. ex. Endl.	0	0	4	18	0	30	0.028	5.7
Maytenus addat (Loes.) Sebsebe	16	2	0			10.8	0.004	0.7
Millettia ferruginea (Hochst.) Bark.	2	11	10	0	0	15.6	0.008	1.5
Myrica salicifolia Hochst. ex A. Rich.	0	9	34	0	0	20	0.013	2.5
Myrsine melanophloeos (L.) R. Br.	2	5	0	0	0	10.8	0.004	0.7
Nuxia congesta R.Br. ex Fresen.	5	9	13		0	15.6	0.008	1.5
Olea europaea L. subsp. cuspidata	4	10	18	7	0	20.5	0.013	2.7
Phoenix reclinata Jacq.	25	20	0	0	0	10.8	0.004	0.7
Podocarpus falcatus (Thunb.) Mirb.	0	5	15	18	22	30.1	0.028	5.8

Prunus africana (Hook. f.) Kalkm.	6	20	7	0	0	15.6	0.008	1.5
Rhus longipes Engl.	18	4	0	0	0	10.8	0.004	0.7
Syzygium guineense (Willd.) DC. subsp. *guineense*	0	4	13	35	20	30.1	0.028	5.8
Total (890)	186	226	248	156	74		0.494	100.0
Basal Area of Trees	0.0012	0.0071	0.0196	0.0385	0.0636	0.1300		
% of Basal Area	0.9	5.5	15.1	29.6	48.9	100.0		
Percentage	20.9	25.4	27.9	17.5	8.3			
