# Supplementary material for: Woody species composition and diversity of riparian vegetation along the Walga River, Southwestern Ethiopia
Source: PLoS One. 2018 Oct 17;13(10):e0204733. doi: 10.1371/journal.pone.0204733 (PMC6192589; doi:10.1371/journal.pone.0204733)
Supplement: S4 Appendix — (PDF) [file pone.0204733.s004.pdf]

Name of Species	Height Classes					No. of Stems	Stem density ha^{-1}	% of Density
	2.5-6.0	6.1-12.0	12.1-18.0	18.1-24.0	>=24.1			
Acacia abyssinica Hochst.	0	30	26	5	0	61	24.4	6.9
Acacia seyal Del.	6	3	0	0	0	9	3.6	1.0
Albizia schimperiana Oliv.	12	8	15	3	0	38	15.2	4.3
Allophylus abyssinicus (Hochst.)	0	6	8	12	2	28	11.2	3.1
Bridelia micrantha (Hochst.) Baill.	0	0	2	4	3	9	3.6	1.0
Cassipourea malosana (Baker) Alston	0	1	6	1	0	8	3.2	0.9
Celtis africana Burm. f.	2	3	6	12	0	23	9.2	2.6
Croton macrostachyus Del.	35	26	8	6	2	77	30.8	8.7
Discopodium penninervium Hochst.	14	3	0	0	0	17	6.8	1.9
Ekebergia capensis Sparrm.	0	0	2	6	10	18	7.2	2.0
Erythrina brucei Schweinf.	3	7	24	8	0	42	16.8	4.7
Euphorbia abyssinica Gmel.	6	14	2	0	0	22	8.8	2.5
Ficus ovata Vahl	5	6	1	0	0	12	4.8	1.3
Ficus sur Forssk.	0	9	16	22	8	55	22	6.2
Ficus thonningii Blume	0	7	0	0	0	7	2.8	0.8
Ficus vasta Forssk.	0	0	11	5	0	16	6.4	1.8
Grewia trichocarpa Hochst. ex A. Rich.	18	2	0	0	0	20	8	2.2
Hagenia abyssinica (Bruce) J.F.Gmelin	2	3	10	2	0	17	6.8	1.9
Juniperus procera Hochst. ex. Endl.	0	0	4	12	6	22	8.8	2.5
Maytenus addat (Loes.) Sebsebe	0	8	10	0	0	18	7.2	2.0
Millettia ferruginea (Hochst.) Bark.	3	7	11	2	0	23	9.2	2.6
Myrica salicifolia Hochst. ex A. Rich.	4	9	30	0	0	43	17.2	4.8
Myrsine melanophloeos (L.) R. Br.	0	0	5	2	0	7	2.8	0.8
Nuxia congesta R.Br. ex Fresen.	0	5	9	13	0	27	10.8	3.0
Olea europaea L. subsp. cuspidata	7	18	10	4	0	39	15.6	4.4
Phoenix reclinata Jacq.	25	17	3	0	0	45	18	5.1
Podocarpus falcatus (Thunb.) Mirb.	0	5	15	18	22	60	24	6.7
Prunus africana (Hook. f.) Kalkm.	0	3	4	8	18	33	13.2	3.7

Rhus longipes Engl.	8	14	0	0	0	22	8.8	2.5
Syzygium guineense (Willd.) DC. subsp. *guineense*	9	24	35	4	0	72	28.8	8.1
Total (890)	159	238	273	149	71	890	356	100.0
Stem density ha^{-1}	63.6	95.2	109.2	59.6	28.4	356		
Percentage	17.8	26.7	30.6	16.7	8.0	100.0		
