# Supplementary material for: Woody species composition and diversity of riparian vegetation along the Walga River, Southwestern Ethiopia
Source: PLoS One. 2018 Oct 17;13(10):e0204733. doi: 10.1371/journal.pone.0204733 (PMC6192589; doi:10.1371/journal.pone.0204733)
Supplement: S5 Appendix — (PDF) [file pone.0204733.s005.pdf]

Kebele	Plots/Quadrats	Altitude	Human impacts	Grazing and browsing	Latitude	Longitude
Chiracha Wember	41	1976	2	4	8^{0}31.752'	37^{0}51.792'
	40	1986	2	3	8^{0}32.076'	37^{0}55.894'
Bedesa Koricha	39	1998	3	2	8^{0}32.459'	37^{0}55.855'
Abado Lemen	38	2008	3	4	832.769'	37^{0}56.050'
Miti Walga	37	2009	2	2	8^{0}33.078'	37^{0}56.210'
	36	2022	3	1	8^{0}33.349'	37^{0}56.210'
	35	2024	3	2	8^{0}33.655'	37^{0}56.329'
	34	2037	3	1	8^{0}33.958'	37^{0}56.409'
	33	2052	3	1	8^{0}34.183'	37^{0}56.455'
	1	2055	3	4	8^{0}34.515'	37^{0}56.862'
	2	2057	1	2	8^{0}34.830'	37^{0}56.981'
	3	2075	2	2	8^{0}35.360'	37^{0}56.116'
	4	2076	1	2	8^{0}35.111'	3756.040'
	5	2088	1	2	8^{0}35.643'	3757.220'
	6	2103	2	3	8^{0}35.913'	3757.252'
	7	2117	3	3	8^{0}36.130'	37^{0}57.200'
	8	2130	3	4	8^{0}36.407'	37^{0}57.197'
	9	2154	3	1	8^{0}36.930'	37^{0}57.214'
	10	2163	2	1	8^{0}37.186'	37^{0}57.162'
	11	2168	3	2	8^{0}37.469'	37^{0}57.076'
	12	2181	3	1	8^{0}37.732'	37^{0}56.987'
	13	2192	3	0	8^{0}38.265'	37^{0}56.983'
Dae wandimtu	14	2203	3	0	8^{0}38.556'	37^{0}57.004'
	15	2212	2	1	8^{0}38.002'	37^{0}56.908'
	16	2222	2	1	8^{0}38.865'	37^{0}57.027'
	17	2231	2	1	8^{0}39.185'	37^{0}57.002'
	18	2251	2	3	8^{0}39.460'	37^{0}56.926'
Sonko Kake	19	2261	3	4	8^{0}39.650'	37^{0}56.639'
	20	2267	3	4	8^{0}39.891'	37^{0}56.430'
	21	2269	2	3	8^{0}40.224'	37^{0}56.313'
	22	2304	3	2	8^{0}40.766'	37^{0}56.009'
Dulele Kore and Fite Wato	23	2319	2	1	8^{0}41.037'	37^{0}55.787'
	24	2359	3	1	8^{0}41.316'	37^{0}55.450'
	25	2433	3	3	8^{0}42.683'	37^{0}54.780'
	26	2455	2	1	8^{0}43.068'	37^{0}54.594'

Azer Qerensa	27	2472	2	0	8^{0}43.271'	37^{0}54.453'
	28	2531	2	1	8^{0}43.614'	37^{0}54.306'
	29	2608	1	0	8^{0}44.118'	37^{0}53.918'
	30	2633	2	1	8^{0}43.749'	37^{0}54.011'
	31	2676	1	0	8^{0}44.485'	37^{0}53.874'
	32	2687	1	1	8^{0}44.671'	37^{0}53.759'
Wando Talfe	50	2710	1	1	8^{0}44. 841'	37^{0}53.659'
	49	2735	0	1	8^{0}45. 457'	37^{0}53.606'
	48	2798	0	0	8^{0}45. 915'	37^{0}53.481'
	47	2824	0	0	8^{0}46. 553'	37^{0}53.617'
Harro Wonchi	46	2888	0	0	8^{0}46.654'	37^{0}52.918'
	45	2996	0	1	8^{0}46.848'	37^{0}52.529'
	44	3022	0	0	8^{0}46.980'	37^{0}52.053'
	43	3069	0	0	8^{0}47.052'	37^{0}51.89'
	42	3078	3	2	8^{0}47.122'	37^{0}51.796'
